# Inflammatory Status of Excavated Pulp Tissue and Internal Root Resorption in Pulpotomized Primary Molars

**DOI:** 10.30476/DENTJODS.2021.88927.1374

**Published:** 2022-09

**Authors:** Roula Berbari, Nahla Nassif, Elia Sfeir

**Affiliations:** 1 Dept. of Pediatric Dentistry and Public Health, Faculty of Dental Medicine, Lebanese University, Beirut, Lebanon

**Keywords:** Cytokines, Internal root resorption, Pulpotomy, Primary molars, Caries

## Abstract

**Statement of the Problem:**

Internal root resorption after pulpotomy is a pathological phenomenon and can lead to early root resorption and subsequent loss of the tooth

**Purpose:**

To assess the relationship between initial inflammatory coronal pulp status in decayed primary molars treated by pulpotomy and internal root resorption after one-year follow-up.

**Materials and Method:**

In this clinical in vivo and in vitro experiment, vital pulpotomies were performed on 50 primary molars from 50 patients aged 5 to 10 years. Coronal pulp was carefully removed followed by hemostasis and placement of a reinforced zinc oxide eugenol over the vital radicular pulp. Enzyme-linked immunosorbent assay (ELISA) assay was done on coronal pulp samples and the level of tumor necrosis factor-alpha (TNF- α) and interlukin-6 (IL-6) was measured. After a 12-month follow-up, periapical radiographs were taken from pulpotomized teeth. Kolmogorov-Smirnov, Chi-square, Kruskal–Wallis, and Mann-Whitney tests were implemented.

**Results:**

11 treated teeth (22%) showed an internal root resorption as diagnosed on X-rays. No significant association was found between TNF- α, IL-6 levels, and pathological root resorption respectively (*p*= 0.953) and (*p*= 0.944). A significant association between age and pathological root resorption was observed (*p*= 0.031). No significant association between remaining dentin thickness and pathological root resorption was established (*p*= 0.346)

**Conclusion:**

There was no association between pro-inflammatory cytokines levels/ TNF-α, IL-6 and internal root resorption following pulpotomy in pediatric patients.

## Introduction

Pulpotomy is a common pulp therapy on carious primary molars [ 1 ]. The coronal pulp is removed and hemostasis is achieved at the level of pulp stumps. The radicular parenchyma, supposedly healthy, is protected with a capping material [ 1 ].

Caries lesions affect the inflammatory status of the pulp and involve the enrolment of immunocompetent and inflammatory blood cells. Pro-inflammatory cytokines like tumor necrosis factor-alpha (TNF-α) and interlukin-6 (IL-6) are released in response to the components of caries-related bacteria. Elevated levels of IL-6 were found in carious deciduous molars with exposed pulp [ 2 ]. The increase in the level of these polypeptides in pulpal blood may help in diagnosis of pulpal inflammation and makes them possible to be used as markers for assessing pulpal status [ 2 - 5 ]. 

The treatments’ success or failures on vital teeth with deep carious lesion are assessed based on radiological and clinical signs and symptoms. Internal root resorption (IRR) after pulpotomy is caused by chronic inflammation of the remaining radicular pulp [ 6 ]. For Smith *et al.* [ 7 ] this process is not a failure since it is not revealing any osseous change or injuries to the permanent successor. For other investigators, IRR is an obvious sign of failure even without clinical manifestations or osseous alterations [ 8 ]. The diagnosis of internal root resorption is mainly based on radiographs with supplementary information gained from history and clinical findings. Contrary to the external root resorption, IRR presents on X-rays a well-defined margin and a symmetrical lesion [ 9 ]. 

The inflammatory role of cytokines in bone resorption and in all disease patterns associated with osteonecrosis is well defined [ 10 ]. Many authors elucidate their impacts on physiological root resorption, on pathological external root resorption during orthodontic tooth movement, and on the process of periodontal tissue remodeling [ 11 - 14 ]. Moreover, cytokines levels are associated with pulp pathogenesis [ 2 ].

This study aims to find a correlation between inflammatory cameral pulp status and IRR after 12 months’ follow-up pulpotomy treatment on carious primary molars. We hypothesized that pathological root resorption after pulpotomy could be related to elevated levels of cytokines (IL6 and TNF-α) on the coronal pulp.

## Materials and Method

This clinical in vivo and in vitro research was carried out at the Department of Pediatric Dentistry and Public Health, Faculty of Dental Medicine with the research platform of the Lebanese University and the approval of its Ethics Committee (protocol #CUMEB/D135/2120-18). An informed consent form was signed by the parents. The sample comprised 50 primary molars selected from 50 healthy patients/ 22 girls and 28 boys, with ages ranging 5 to 10 years.

Teeth were selected according to pre-established criteria. The inclusion criteria were: (1) vital primary molars with deep carious lesions with no symptoms of advanced pulpal inflammation or evidence of radicular pathology, (2) remaining dentin thickness (RDT) measured on X-rays from the deepest point of a carious lesion to the nearest point of the pulp, ranging between 0.3mm≤RDT≤1.0 mm, (3) presence of at least two-thirds of the root length, (4) restorable tooth, and (5) contralateral healthy deciduous molar allocated to the control group.

The exclusion criteria were: (1) presence of a fistula, a radicular radiolucency, a pulp calcification, or a pathological root resorption, and (2) uncontrolled bleeding after pulp amputation exceeding 5 minutes. Before the preoperative radiograph, a quick-setting silicone paste (Occlufast Rock-Silicone-A-Zhermack, Italy) was injected around a sensor holder (Kwik-bite/Art.No.270-Kerr, Switzerland) to record the occlusion on each side. 

All periapical radiographs were taken with a paralleling technique using a digital sensor in conjunction with a thyroid shield apron.

The radiographs were processed in a digital reader (VistaScan Mini Plus: Dürr Dental, Germany). 

Vital pulpotomy was performed. The access of the pulp chamber was done with a sterile round diamond bur under physiological saline irrigation. Coronal pulp, usually eliminated in drilling operation, was carefully detached with a sharpened excavator. The tissue was then placed in a sterile tube containing 1 ml of reducing medium (Thioglycolate: SIGMA) and sent directly to the laboratory. Adequate hemostasis was achieved by gentle pressure with a sterile cotton pellet soaked in physiological saline (sodium chloride 0.9%) against the canal orifices for 5 minutes. Zinc oxide-eugenol with polymer reinforcement (IRM® /Caulk Dentsply) was placed. The molars were immediately restored with pediatric stainless-steel crown (3M ESPE) followed by postoperative periapical digital X-rays. 

## Results

Follow-up examinations of the pulpotomized teeth and healthy ones were carried out after 12 months. The collected sample was transferred into a single well of an IL-6/TNF-α assay plate supplied by the manufacturer (R&D System Bio‑Techne) and the Enzyme-linked immunosorbent assay (ELISA) was applied.

Statistical analysis was conducted using IBM SPSS version 24 (IBM Corp., Armonk, N.Y., USA). Descriptive statistics were presented as mean ± standard deviation for continuous variables and as frequencies and percentages for categorical variables. Kolmogorov-Smirnov, Chi-square, Kruskal–Wallis, and Mann-Whitney tests were done. Level of significance was set below *p*= 0.05. Results

A total of 50 primary molars /19 second primary molars and 31 first primary molars, with deep dentinal caries and a value of remaining dentin thickness ranged from 0.3mm to 1mm, requiring vital pulpotomy, were included in this study ( Table 1). The distribution of patient age and gender is outlined in Table 2. From 50 selected patients aged between 5 and 10, mean age=6.8 years ( Table 1), 22 were female and 28 males (Chi-square test, *p*= 0.480), ( Table 2) and distributed as follow: 6 patients were 5 years old, 22 between 6 and 8 years and 22 from 8 to 10 years (Chi-square test, *p*= 0.006), ( Table 2).

**Table 1:** Descriptive statistics of continuous variables

	Age	RDT	TNF-α	IL-6
Mean	6.80	0.616	1159.40	1007.56
Median	6.75	0.600	525.00	487.50
Mode	7	0.4	17	19
Standard Deviation	1.265	.2189	1332.324	1149.244
Minimum	5	0.3	13	11
Maximum	10	1.0	3950	3620

TNF- α : Tumor necrosis factor-alpha, IL-6: Interlukin-6, RDT: Remaining dentin thickness

**Table 2:** Repartition of the categorical variables by age groups, gender

		Frequency	Percent	*Chi-square test*
Age groups	5	6	12.0	*p*= 0.006
	6-7	22	44.0	
	8-10	22	44.0	
Gender	F	22	44.0	*p*= 0.480
	M	28	56.0	
TNF-α	0-500	24	48.0	
	501-1000	9	18.0	
	>=1001	17	34.0	
IL-6	0-500	26	52.0	
	501-1000	8	16.0	
	>=1001	16	32.0	
IRR	Absence	39	78.0	
	Presence	11	22.0	
Total		50	100.0	

TNF- α: Tumor necrosis factor-alpha, IL-6: Interlukin-6 and IRR: Internal root resorption

In 50 collected pulp samples, level of cytokines after quantification ranged from 13 pg/ml to 3950 pg/ml for TNF-α and from 11 pg/ml to 3620 pg/ml for IL-6 with a mean value of 1159.40 and 1007.56 respectively ( Table 1). Moreover, almost 48% presented a low level of TNF-α (0-500pg/ml), 18% presented a level between (501-1000pg/ml), and 34% have a level ≥ 1001 pg/ml ( Table 2). For the IL-6: 52% were at a low level (0-500 pg/ml), 16% were between 501-1000 pg/ml, and 32% were≥ 1001 pg/ml ( Table 2). No data obtained followed a normal distribution (Kolmogorov-Smirnov test, *p*≤ 0.001). After one year of follow-up, 11 treated teeth (22%) showed internal root resorption ( Table 2). There was no significant correlation between inflammatory cytokines (TNF-α and IL-6) and pathological root resorption (respectively Mann-Whitney test, *p*= 0.953 and *p*= 0.944), ( Table 3). No significant association between remaining dentin thickness and pathological root resorption was established (Mann-Whitney test, *p*= 0.346), ( Table 3). A significant relationship between age and pathological root resorption was noted. Internal root resorption was positively correlated with age (Mann-Whitney test, *p*= 0.031), ( Table 3). 

**Table 3:** Relationships between internal root resorption (IRR) with: age

	IRR	Frequency	Rank	Mean Rank	Statistics	*p*
Age	Absence	39	23.17	903.50	123.500	0.031*
	Presence	11	33.77	371.50		
	Total	50				
RDT	Absence	39	24.49	955.00	175.000	0.346
	Presence	11	29.09	320.00		
	Total	50				
TNF-α	Absence	39	25.56	997.00	212.000	0.953
	Presence	11	25.27	278.00		
	Total	50				
IL-6	Absence	39	25.42	991.50	211.500	0.944
	Presence	11	25.77	283.50		
	Total	50				

* Denotes significance at *p* > 0.05

RDT: Remaining dentin thickness, TNF-a: Tumor necrosis factor-alpha and IL-6: Interlukin-6

## Discussion

In this research, the inflammatory coronal pulp status was appraised as a factor affecting the internal root resorption. Assessments were made based on the levels of inflammatory results as well as periapical radiographic evaluation.

The proximity of carious lesions to the pulp was measured on preoperative X-rays according to the technique applied by Berbari *et al.* [ 15 ]. To detect and to measure the pro-inflammatory cytokines, the assay by the high sensitivity ELISA technique was considered as a validated method [ 3 ]. The number chosen for our samples (n= 50) was enough to carry out the statistical analysis as well to determine the significance of the results [ 16 ]. To minimize the patient’s exposure, 12 months of follow-up seemed an acceptable period to evaluate the treatment’s outcomes [ 17 ].

To rule out the factors that could interfere with the results, a well-established methodology was carried out by a single operator. Moreover, copious irrigation was done to avoid tissues’ necrosis and to enhance the healing capacity of the residual vital pulp. After pulpal treatment, a stainless-steel crown was immediately well-sealed to prevent leakage and thereby bacterial infiltration and inflammation of the remaining pulp and possible occurrence of IRR [ 18 - 20 ].

The American Academy of Pediatric Dentistry [ 20 ] recommended pulpotomy in primary tooth with extensive caries. For Ghaderi *et al.* [ 21 ] caries extension measured on radiographs could be a potential diagnostic tool to pulpotomy treatment. In this study, the inclusion criteria required a mean value of remaining dentin thickness between 0.3mm and 1.0mm ( Table 4). According to Berbari *et al.* [ 16 ], this element may prove itself as an additional indicator for pulpotomy.

**Table 4:** Collected data, for each sample table shows: Gender, Age, RDT expressed in mm, Cytokine levels, IL-6, TNF- α expressed in pg/ml and IRR (+means presence of IRR and– means absence of IRR)

Sample #	Gender	Age	RDT (mm)	IL-6 (pg/ml)	TNF-α (pg/ml)	IRR
1	F	5	.0.7	1420	1580	-
2	F	8	.0.8	32	40	-
3	F	9	1	1880	2250	-
4	F	8	.0.3	450	520	-
5	M	8	.0.6	11	16	-
6	F	6	.0.4	1980	2840	-
7	F	7	.0.3	14	19	-
8	M	6	.0.4	15	17	-
9	M	6	.0.4	2940	3840	-
10	M	6	.0.6	29	35	-
11	F	7	.0.5	23	22	-
12	M	7	.0.4	18	17	+
13	M	8	.0.4	890	1220	-
14	M	8	.0.4	55	95	-
15	F	6.5	.0.4	190	160	-
16	M	6	.0.4	12	14	-
17	F	8	.0.5	2100	2920	-
18	M	8	.0.9	189	145	-
19	F	7	.0.5	1750	1890	+
20	M	6	.0.5	14	13	-
21	M	5	.0.7	795	1100	-
22	M	8	1	19	25	-
23	F	10	.0.9	2850	3420	+
24	F	8	.0.7	12	14	+
25	F	8	.0.8	18	22	-
26	F	8	..0.5	1524	1100	-
27	M	6.5	0.9	3620	2980	-
28	M	9	1	20	21	-
29	M	5	.0.7	2970	3950	+
30	M	5	.0.8	3100	3570	-
31	M	6	.0.4	1290	1720	-
32	F	5	.0.6	19	22	-
33	F	8	.0.4	2420	2200	-
34	F	8	.0.7	17	19	-
35	M	8	1	1320	1240	+
36	M	6	.0.4	3460	2970	-
37	M	6	.0.9	1795	2650	-
38	F	8	.0.5	2890	3790	-
39	M	6.5	.0.5	890	1090	-
40	M	9	.0.4	85	110	+
41	F	6	.0.6	820	790	+
42	M	6	.0.4	480	530	-
43	M	6	.0.7	495	550	-
44	M	9	1	520	462	+
45	M	5	.0.8	41	55	-
46	M	8	.0.9	2690	2890	-
47	M	6	.0.6	19	17	-
48	F	8	.0.8	22	20	+
49	F	6.5	.0.4	190	160	+
50	F	6	.0.4	1975	2830	-

F= Female, M= Male, RDT: Remaining Dentin thickness, IL-6: Interlukin-6, TNF- α: Tumor necrosis factor-alpha, IRR: Internal root resorption

High levels of cytokines can be an indicator of preoperative pulpal inflammation and a main cause of failure after pulpotomy [ 2 , 22 ]. Ozdemir *et al.* [ 2 ] detected high level of cytokines in pulp samples among unsuccessful pulpotomy. Likewise, Waterhouse *et al.* [ 23 ] reported a positive correlation between prostaglandin E2 (PGE2) a mediator of inflammation, and radiological outcome following vital pulp therapy. In this study, although we detected a high level of cytokines in four cases with IRR (samples #19, #23, #29, #35) ( Table 4), no-correlation was found between inflammatory cytokines/ TNF- α and IL-6 and pathological root resorption. In the same line, very high levels of TNF-α and IL-6 in another samples, did not lead to pathological root resorption (samples #9, #36, #38), ( Table 4). Conversely, with a low level of cytokines, some cases presented a pathological root resorption (samples #12, #24, #48), ( Table 4). These contrasting results could be justified by many different etiological factors such as an erroneous pulp diagnosis before treatment decision [ 24 ]. In fact, establishing a correct diagnosis is essential to obtain high success rates in pulpotomy treatment. Assessing the pulpal status of primary teeth can be difficult since children are not capable to give detailed history of symptoms. Moreover, the exact pulp diagnosis could not be consistent with the severity of the symptoms [ 25 ].

During pulpotomy, pulp diagnosis is based on radiological evaluation and clinical signs. The color, the aspect and the intensity of pulp bleeding were considered as predictable factors for pulpotomy success [ 21 , 26 ]. Conversely, recent studies showed no direct correlation between successful hemorrhage control and radicular pulp inflammation [ 27 ]. In the study of Fuks *et al.* [ 28 ], the intraoperative diagnosis/ bleeding time and color, although clinically reliable, did not precisely determine the condition of the pulp tissue. In this research, in line with the results of this study, elevated level of cytokines was observed in some pulp samples (#27, #30, #36) even though all the pulpotomies were performed within the hemostasis time (Table 4). 

The formation of a blood clot after pulpotomy, followed by a chronic inflammation of the residual pulp, might be an additional causal factor for internal root resorption [ 29 - 30 ]. The placement of the material over a bleeding pulp or a clinically observable blood clot is favorable for microbial development. According to Shröder [ 30 ], extra-pulpal blood clot between the wound surface and the dressing materiel would increase the initial inflammatory response originated by a pulpotomy agent, due to the chemotactic effect of the polymorphonuclear leukocytes. To dissolve any extra-pulpal blood clot, sodium hypochlorite was suggested [ 31 ]. In the current study, the hemostasis was achieved by a cotton pellet soaked with sterile saline. Thus, could be one of the main reasons of internal root resorption.

The wound dressing material is an important factor affecting the outcome of the pulpotomy. The ideal capping material should be bactericidal, harmless to cells, and promoting the healing of pulp tissue without interfering with the physiological root resorption [ 32 - 33 ]. According to Ratnakumari and Thomas [ 32 ], the pulp response is the best criterion to judge the performance of a capping agent. To evaluate the relationship between cytokines and pulpotomy agents, Lourenço Neto *et al.* [ 34 ] remarked that clinical and radiographic successes are not consistent with the true state of pulp health. So far, the presence of an ideal pulp dressing material is not evident [ 35 ]. In this current study, zinc oxide-eugenol with polymer reinforcement was used knowing to have decreased irritating effects of eugenol and the presence of polymethylmethacrylate [ 36 ]. This product was applied without any fixative, preservative or astringent agent, while maintaining the integrity of the root pulp. In the study of Hui-Derksen *et al.* [ 37 ] the overall success rates were obtained after vital pulp pulpotomy with reinforced zinc oxide-eugenol made this latter a reliable procedure for non-chronically inflamed primary molars. Moreover, a low rate of internal resorption (~2%) was observed [ 37 ].

In the current research, internal root resorption was positively correlated with age (Table 3). Controversial results from previous studies suggest that no substantial changes occur in the structural features or in vital functions of primary teeth pulp during the aging period [ 38 - 40 ]. The results of the study of Sönmez and Durutürk [ 8 ] showed that IRR was not affected by physiological root resorption. 

The present study showed no significant association between remaining dentin thickness and pathological root resorption (*p*= 0.346), (Table 3). In fact, a lesion very close to the pulp does not induce necessarily a pathological root resorption (samples #4, #7), (Table 4). On the other hand, a pathological resorption was observed in shallower caries (samples #35, #44), (Table 4). When performing a pulpotomy, regardless of the numerical value of the caries depth, many other factors should be considered to avoid treatment’s failure. The stage of caries lesion activity, the thickness and degree of calcification of the remaining dentine, the nature and aspect of the residual dentin, and especially the reactionary dentin or sclerotic dentin should be carefully examined [ 16 , 21 ]. 

This original article showed for practitioners the relationship between initial inflammatory coronal pulp status and internal root resorption. When performing pulpotomy in carious primary molars, dentists should always keep in mind all factors leading to pathological root resorption even if this latter was not clinically observed. This awareness saves them from being tempted to think of a causal relation. Moreover, this subject opens for researchers the opportunities to deal with such topic again.

The current study was not without limitations; the overlapping of the dental and bone structures in maxilla made radiological observation difficult and some inflammatory cytokines were not studied. An increase in sample size and follow up period would lead to results that are more accurate. Further research is warranted to study the association between immune pulp status and internal root resorption.

## Conclusion

In this research, TNF- α and IL-6 levels were evaluated as factors affecting the outcomes of pulpotomy treatment. The study found no association between inflammatory coronal pulp status and internal root resorption following pulpotomy. Internal root resorption diagnosed on radiography after pulpotomy could be unnoticed clinically. 

## Conflicts of Interest

 The authors declare that they have no conflict of interest. 
